# A virus–target host proteins recognition method based on integrated complexes data and seed extension

**DOI:** 10.1186/s12859-022-04792-x

**Published:** 2022-06-28

**Authors:** Shengrong Xia, Yingchun Xia, Chulei Xiang, Hui Wang, Chao Wang, Jin He, Guolong Shi, Lichuan Gu

**Affiliations:** 1 School of Information and Computer, Anhui Agricultural University, Hefei, 230036 Anhui China; 2 Key Laboratory of Agricultural Electronic Commerce, Ministry of Agriculture, Hefei, 230036 China; 3 Institute of Intelligent Agriculture, Anhui Agricultural University, Hefei, 230036 China

**Keywords:** Virus–target protein, Host protein, Protein complexes recognition, Protein–protein interaction network

## Abstract

**Background:**

Target drugs play an important role in the clinical treatment of virus diseases. Virus-encoded proteins are widely used as targets for target drugs. However, they cannot cope with the drug resistance caused by a mutated virus and ignore the importance of host proteins for virus replication. Some methods use interactions between viruses and their host proteins to predict potential virus–target host proteins, which are less susceptible to mutated viruses. However, these methods only consider the network topology between the virus and the host proteins, ignoring the influences of protein complexes. Therefore, we introduce protein complexes that are less susceptible to drug resistance of mutated viruses, which helps recognize the unknown virus–target host proteins and reduce the cost of disease treatment.

**Results:**

Since protein complexes contain virus–target host proteins, it is reasonable to predict virus–target human proteins from the perspective of the protein complexes. We propose a coverage clustering-core-subsidiary protein complex recognition method named CCA-SE that integrates the known virus–target host proteins, the human protein–protein interaction network, and the known human protein complexes. The proposed method aims to obtain the potential unknown virus–target human host proteins. We list part of the targets after proving our results effectively in enrichment experiments.

**Conclusions:**

Our proposed CCA-SE method consists of two parts: one is CCA, which is to recognize protein complexes, and the other is SE, which is to select seed nodes as the core of protein complexes by using seed expansion. The experimental results validate that CCA-SE achieves efficient recognition of the virus–target host proteins.

**Supplementary Information:**

The online version contains supplementary material available at 10.1186/s12859-022-04792-x.

## Introduction

The novel coronavirus (2019-nCov for short) outbroke at the end of 2019, and has been causing varying degrees of disease symptoms in people of different ages, such as gastrointestinal symptoms in children while other ages experienced pneumonia, stroke, and blood clots [1]. 2019-nCov belongs to the $\beta$ coronavirus, which is a single-stranded RNA virus. In addition to the discovery of 2019-nCov in coronavirus, six human coronaviruses have been found and studied in depth. There are four types of $\beta$ coronavirus group. In 2002, there were 8000 cases of severe acute respiratory syndrome coronavirus (SARS-CoV) worldwide, and the mortality rate was about 10%. The Middle East respiratory syndrome coronavirus (MERS-CoV) produced in 2012 had a higher fatality rate of 36% [2, 3]. The other two beta-viruses (HCov-HKU1 and HCov-OC43) are related to respiratory diseases, but with low incidence. There are two kinds of $\alpha$ coronavirus groups [4], such as HCoV-229E in 2000, but the pathogenicity and mild symptoms. The HCov-NL63 virus, found in the Netherlands in 2004, had little damage to humans and can be ignored. 2019-nCov can be transmitted by face-to-face communication or direct contact of faces [5]. For patients infected with 2019-nCov, there are few drugs available to treat the virus, such as Lopinavir, Remdesivir, and symptom-based treatment [6].

Proteins with similar functions tend to form protein complexes and work together, as do virus proteins. Therefore, it is very important to recognize protein complexes in predicting virus–target host proteins. Detecting complexes from Protein–Protein Interaction (PPI) has become an important research field in proteomics. Many graph clustering algorithms use it to find community structures in networks, and recognize protein complexes. Nepusz et al. [7] proposed ClusterONE, which for detecting potentially overlapping protein complexes from PPI data. Liu et al. developed CMC [8] to discover complexes from a weighted PPI network. Min Wu et al. presented COACH [9] which detected protein complexes in two stages. COACH first detects protein-complex cores as the “hearts” of protein complexes and then includes attachments into these cores to form biologically meaningful structures.

According to the World Health Organization, over 17 million people die of infectious diseases every year. It is necessary to identify interactions between viruses and human proteins in virus disease treatment. Ho-Joon Lee [10] presented an approach to predict virus–host PPI by multi-label machine learning classifiers of random forests and XGBoost using amino acid composition profiles of virus and human proteins, which predict that histone H2A components are targeted by multiple 2019-nCov proteins. Jack Lanchantin [11] proposed DeepVHPPI, combining a self-attention-based transformer architecture and a transfer learning training strategy to predict interactions between human proteins and virus proteins that have novel sequence patterns, achieving great results in predicting virus–human protein interactions for H1N1 and Ebola. Babak Khorsand [12] adopted ensemble learning methods to predicting PPI between human proteins and Alphainfluenzavirus proteins, he extracted several features from physicochemical properties of amino acids, combined with different centralities of human PPI.

Studying PPI between the new virus and its known host proteins can accurately predict the virus. Fatma-Elzahraa Eid [13] proposed DeNovo, a sequence-based negative sampling and machine learning framework, he learns from the PPI of different viruses and predicts a new virus using shared host proteins. Experiments show that this method can better predict the PPI of human virus infection. Dyer et al. [14] proposed a method to predict the physical interaction between human proteins and HIV proteins based on a variety of features, such as protein domains, sequence information, and the properties of human proteins in human PPI networks. Zahiri predicted the HIV1–human PPI network [15]. Ray predicted HCV–human PPI network [16]. Chen revealed the west Nile virus–human PPI network [17].

However, most existing methods often ignore the inherent biological structure of the complexes, and many only consider the structure as dense subgraphs. Few of them consider the modularity of PPI, let alone search for potential virus–target proteins from protein complexes, such as Babak Khorsand [18]. Here we adopt CCA to recognize protein complexes, showing the feasibility of seed selection, and then we used CCA-SE to predict potential virus–target human proteins. At first, a network representation learning technique called node2vec is utilized to learn a dense vector for each vertex to represent the topological information. Secondly, based on edge clustering coefficient (ECC) and degree, we introduce a new seed selection strategy, and the core structure of protein complexes is detected based on SE. Then according to network topology, we design a fitness function to identify protein complexes with various densities and modularity. At last, we apply CCA-SE to predict the 2019-nCov-target proteins, which play a fundamental role in detecting virus drug targets.

## Result

### Principle of the CCA-SE method

**Fig. 1:**
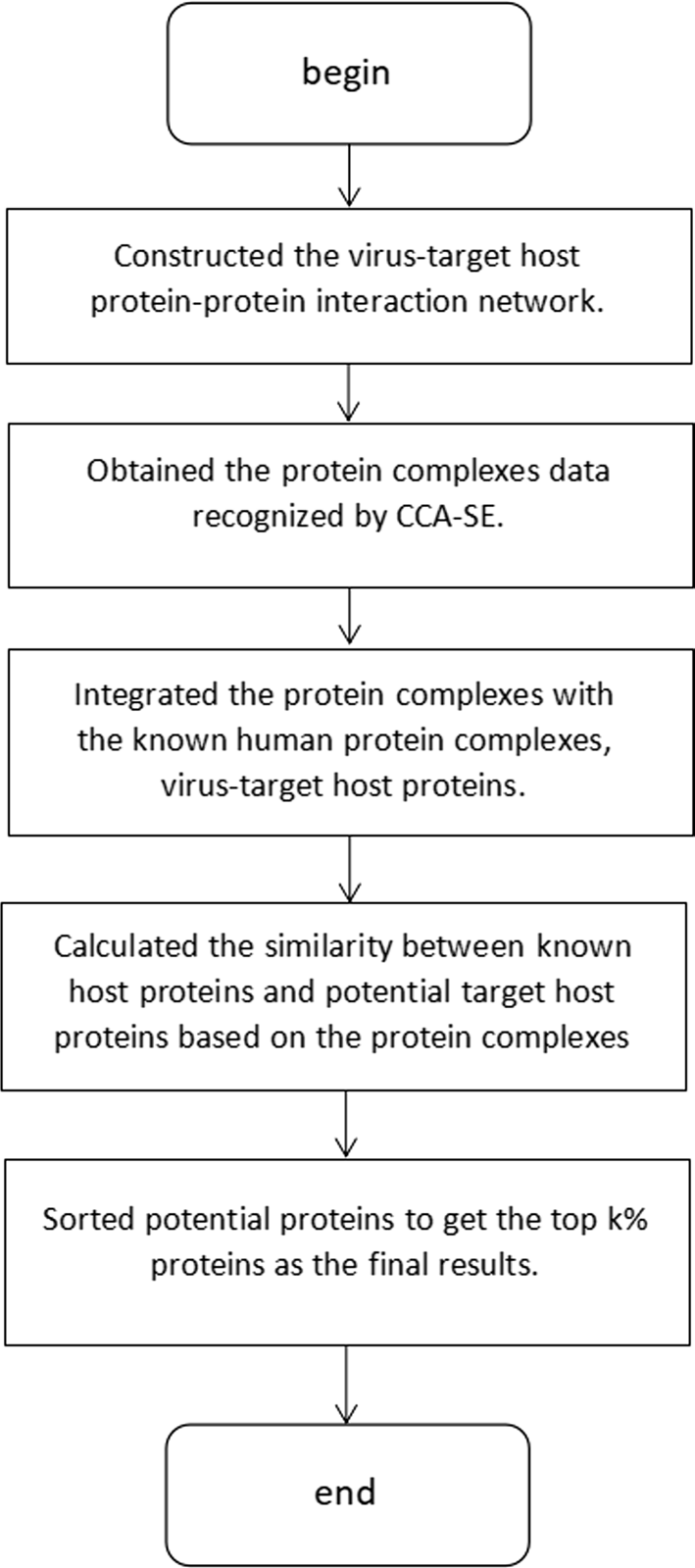
Process of CCA-SE

We developed the CCA-SE method based on the principle of coverage clustering-core-subsidiary structure, which contained two parts, CCA was used to recognizing protein complexes, and SE was applied to select seed nodes. Especially, We demonstrated a new selection strategy for seed nodes. We clustered seed nodes to obtain the nuclear structure of protein complexes. Then, based on the density and modularity, we expanded the core structure and formed protein complexes. Next, integrate downloaded complexes data with our results. At last, we calculated the similarity between unknown human proteins and known virus–target proteins in the same protein complexes. We defined a scoring function, which was helpful in getting potential virus–target human proteins. Figure 1 shows the algorithm.

#### Construction of virus–host PPI network

We constructed a virus–target human PPI network based on the human protein interaction database HIPPIE and the human protein dataset of 2019-nCov infection. Researchers often regard direct neighbor proteins as potential host proteins in some typical virus–host proteins research [19]. Therefore, G = (V, E) represents human PPI. V includes not only host proteins attached by the 2019-nCov directly but also direct neighbors of those host proteins. E shows human PPI. After removing noisy data, we got 2308 human proteins. We reported details in “Methods” section.

Node2Vec [20] not only makes similar nodes closer in the vector space but also retains network structure, captures the diversity connection between nodes. We embedded Node2Vec into the PPI network to extract hidden information and used a low-dimensional vector to represent each node.

**Fig. 2:**
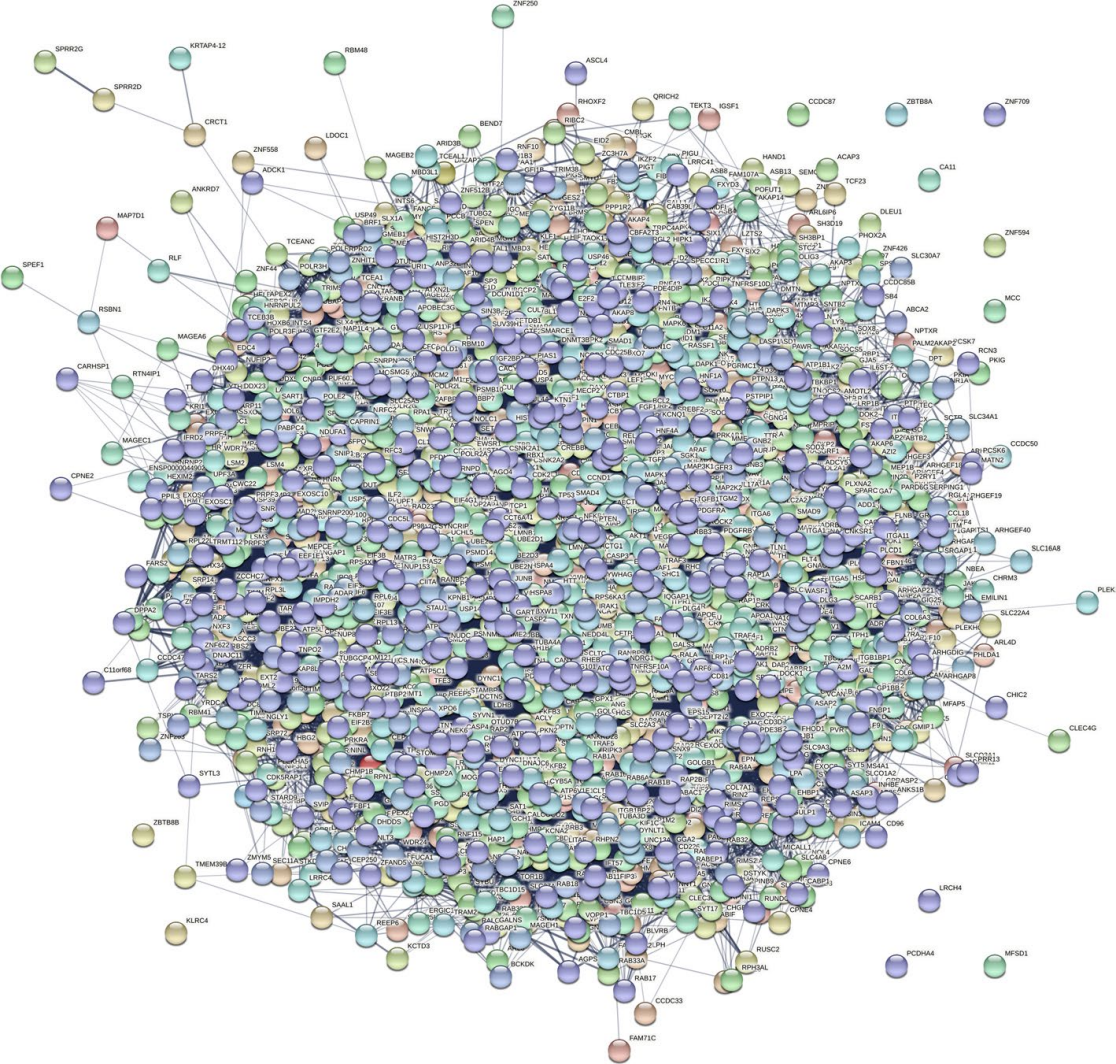
An illumination of virus–host PPI Network

We extracted the hidden layer weight of PPI and demonstrated each protein node with a 128-dimensional vector [21]. In Fig. 2, nodes included not only the proteins attached by virus but also those known interacted with virus–host proteins directly.

#### Selection of seed nodes

In a real protein network, many protein complexes are overlapped and share multiple nodes, so that the core nodes and the overlapped nodes cannot be distinguished simply by degree [22]. Therefore we use two topological properties, ECC and nodes degree, which can evaluate the importance of nodes. The score of each protein is obtained by the sum of degree and ECC. According to an existing work [9], if the score of a protein node is greater than the average score, we consider it as a seed protein that would achieve the best results. The scoring function for each protein *u* is defined in Eq. (1). We proved Eq. (1) feasibility in Table 1.

1 $$\begin{aligned} score(u) = \frac{{sumEEC(u)}}{{\deg ree(u)}}\ \end{aligned}$$

where sum$_{EEC}$(*u*) indicates summation of ECC and degree(*u*) indicates degree of node *u*. Equation (1) considers network topological structure and reduces the overlapped nodes that include the sum of ECC by dividing each node degree. Meanwhile, it avoids overlapped nodes mistaken as seed nodes and improves seed selection accuracy.

**Table 1:** Effects in different scores

Score	Seed	Precision	Recall	F-score
0.1398	1035	0.9317	0.6399	0.7587
0.1498	982	0.9350	0.6352	0.7565
0.1598	939	0.9390	0.6381	0.7598
0.1698	868	0.9414	0.6406	0.7624
0.1798	821	0.9383	0.6334	0.7563
0.1898	781	0.9272	0.6119	0.7373
0.1998	726	0.9399	0.6078	0.7382

We formed Table 1 based on our results. “Score” means the standard score when we choose seed nodes. “Seed” means the number of seeds. We use 0.1698 as the seed selection standard also is the average score. As we can see from Table 1, whether the score is higher than average or lower, their precision, recall, and F-score are not good as the average score’s effect.

We can use Gene Ontology (GO) database to predict and analyze gene function. Usually, a gene or gene product is annotated by one or more GO terms. We can calculate the similarity between genes to analyze and predict gene function. The traditional coverage clustering algorithms (CCA) [23] are less time-consuming, handle large amounts of data, and have no overlapping region among obtained clusters. However, these methods would generate much false positive data without a reasonable clustering radius. Therefore, we use GO function similarity to obtain a reasonable radius and improve clustering accuracy. The clustering radius is computed as follows.

2 $$\begin{aligned} d_x = \frac{{r_x}}{{1 + GO_{similarity}}} \end{aligned}$$

3 $$\begin{aligned} radius = \frac{{\sum \nolimits _{x \in D} {d_x} }}{{\sum \nolimits _{x \in D} 1 }}\ \end{aligned}$$

We denote rx, the distance of all unlearned seed nodes to the clustering center based on the Euclidean distance. We redefine the distance as shown in Eq. (2). The clustering radius of each final cluster is obtained by Eq. (3). D indicates the seed nodes that have not learned yet.

It shows that in PPI networks, high-density subgraphs incline to form protein complexes [24]. Also, in a subgraph, if the internal weight is much greater than the external weight, it is more likely to form protein complexes. Therefore, density [25] and modularity [26] are two important factors, determining whether the subgraph could form protein complexes or not. We demonstrate a new method to evaluate protein complexes.

4 $$\begin{aligned} score(c_u) = t * density(c_u) + (1 - t) * \bmod ularity(c_u)\ \end{aligned}$$

5 $$\begin{aligned} density(c_u) = \frac{{2 * |E_{c_u}|}}{{|V_{c_u}|(V_{c_u} - 1)}}\ \end{aligned}$$

6 $$\begin{aligned} \bmod ularity(c_u) = \frac{{\deg ree_{in}(c_u) - \deg ree_{out}(c_u)}}{{\deg ree_{in}(c_u) + \deg ree_{out}(c_u)}}\ \end{aligned}$$

degree$_{in}$(c$_u$) is the sum of internal edges of the complexes, degree$_{out}$(c$_u$) is the sum number of other edges connected with the complexes, E$_{Cu}$ is the number of all edges in the complexes, and V$_{Cu}$ is the number of all nodes in the complexes. Moreover, we use a parameter *t* to balance the weight of subgraph modularity and subgraph density, as shown in Eq. (4).

#### Obtaining potential virus–host proteins

We obtain a set of protein complexes cores. To form complete protein complexes, we add subsidiary structures to each core with the following steps and name them SE: (i) The first-order neighbor nodes of each protein complexes core are obtained from the network diagram as candidate proteins of the complexes. (ii) Calculating the sum of functional similarity between the core and their neighbor nodes based on edge weight. (iii) ranking nodes according to the functional similarity score, completing protein complexes by adding affiliated nodes. The local score of the protein complexes calculates when a new node adds. (iv) Repeating (iii) until no more nodes can improve the local score, then terminating the expansion of the protein complexes core.

Protein complexes are molecular polymers that participate in the same functional region at the same time and space and have direct or indirect effects [27]. Studies have shown that the network is modular, and proteins in the same complexes are more likely to undertake the same life activity or in the same pathway [28]. At the same time, the more host proteins contained by the complexes indicate that the complexes are more likely to participate in life activities such as virus replication [29].

We obtained 255 protein complexes on the human PPI network dataset. Considering incomplete data, we downloaded all human complexes data from the CORUM database, including 1948 protein complexes. Then we integrated them with our results, choosing protein complexes data with nodes than three and known host proteins while deleting the redundant data. Therefore we got 455 human complexes, with complexes that contain more than two known proteins having sixty. The more known host proteins contained in the complexes, the more likely it participate in viral survival and reproduction. Since the proteins phenotypes in the same protein complexes are similar, the remaining proteins may also become the proteins required for virus replication.

Table 2 lists some protein complexes, in which Complex_size represents the size of the module, Host_protein is the host protein of the virus, and Complexes is the collection of all proteins.

**Table 2:** Partial protein complex data

ID	Complex_size	Host_protein	Complex
1	4	P67870; P19784;	P09429; P19784; P67870; P68400;
2	5	Q15370; P62877; Q15369; Q13617;	P40337; Q15370; P62877; Q15369; Q13617;
		Q15369; Q13617;	Q15369; Q13617;
3	5	Q15370; Q15369;	Q15369; Q15370; Q93034;
			Q9UBF6; Q9Y576;
4	7	Q92769;	O00422; O75446; Q09028; Q13547;
			Q16576; Q92769; Q96ST3;
5	3	P13861;	P13861; P24588; Q08209;
6	5	Q9UHD2;	P18124; P08238; Q04206; Q9UHD2;
			Q00653;
7	3	Q86VM9;	Q86VM9; Q9BXB5; Q15287;
8	5	P11940; O75534;	P11940; O75534; Q16549; Q14103;
			O60506;
9	16	O43633; Q70EL1;	Q9BY43; O95630; Q9UN37; Q70EL1;
			Q96FZ7; Q96CF2; Q9NZZ3; Q9H444;
			Q70EL1; Q9HD42; O43633; O75351;
			Q9UN37; Q7LBR1; Q9H444; Q9BY43;
10	9	O60885; Q7L2J0;	O94992; O60563; O60583; O60563;
			P06400; O94992; O60885; P50750; Q7L2J0;

Based on Table 2, we can speculate that the unknown human proteins in the same complexes are closely related to the known virus–host proteins. The table also shows that not only other proteins in the module are related to the virus, but also the module itself is related to the virus, which jointly completes some biological functions and promotes the reproduction of the virus. Therefore, these remaining proteins are potential virus–target proteins we need.

The above descriptions show that each of the complexes contains virus–target host proteins and is more or less associated with the virus. Then we use the protein complexes and Node2Vec to calculate the similarity between the rest of the unknown proteins and known host proteins, then score them as potential target proteins. The score computes as follows.

7 $$\begin{aligned} score_c = w_c * sim_c(complex)\ \end{aligned}$$

w$_c$ indicates the proportion of known host proteins in the complexes to which potential target protein *C* belongs. n$_c$ indicates the number of known proteins in the complexes. Let *N* indicates the total number of proteins in the complexes. w$_c$ in Eq. (8) can be obtained as follows.

8 $$\begin{aligned} w_c = \frac{{n_c}}{N}\ \end{aligned}$$

The second term in Eq. (7) indicates the sum of the similarities between target protein *C* and the known proteins in its complexes. The formula is as follows.

9 $$\begin{aligned} sim_c(complex) = \sum \limits _{i = 1}^n {sim(c,P_i)} \ \end{aligned}$$

*P*$_i$ indicates each known virus–target protein in the complexes of candidate protein *C*, in which the complexes in the Equation select the complexes corresponding to the maximum value in Eq. (8).

## Methods

### Statistical model

We define the functional similarity between the two interacting proteins *a* and *b* as GO$_{similarity}$, and as follows.

10 $$\begin{aligned} GO_{similarity}(a,b) = |GO_{sum}(a) \cap GO_{sum}(b)|\ \end{aligned}$$

Based on the definition of protein functional similarity, the adjacency matrix A$_{ij }$ of graph *G* can express as follows.

11 $$\begin{aligned} A_{ij} = \left\{ {_{0,\quad otherwise}^{e_{ij}(i,j \in E)}} \right. \ \end{aligned}$$

e$$_{ij}$$ equals GO$$_{similarity}$$.

### Dataset

Based on the latest study of the interaction map of 2019-nCov virus–host protein [30], we obtained 332 high reliability 2019-nCov human PPI data. We mapped the proteins with the Uniprot ID through the Uniprot database and got 256 key host factors in our experiments. To reduce the impact of data redundancy on results, we used the human PPI data in HIPPIE, which integrates interactive data of multiple databases, including MINT, HPRD, and BioGrid. The HIPPIE is the most commonly used PPI database. Therefore, we download 65,536 PPI data from the HIPPIE database, involving 11,564 human proteins.

### Weighting protein networks by GO annotations

GO is an internationally standardized gene function classification system. It consists of a predefined set of GO terms, which can limit and describe the function of gene products. GO terms provide the logical structure and correlation of biological processes and classify biological process (BP), molecular function (MF), and cellular component (CC). GO annotations [31] are responsible for describing GO terms function. We use G = (V, E) to represent the proteins network, V is the set of proteins, and E represents the set of protein–protein interactions. The specific steps are as follows: (i) We assume that protein *a* contains *N* GO annotation sets on BP.

12 $$\begin{aligned} GO_{BP}(a) = \left\{ {go_1,go_2, \dots ,go_n} \right\} \ \end{aligned}$$

*M* GO annotation sets on MF.

13 $$\begin{aligned} GO_{MF}(a) = \left\{ {go_1,go_2, \dots ,go_m} \right\} \ \end{aligned}$$

*K* GO annotation sets on CC.

14 $$\begin{aligned} GO_{CC}(a) = \left\{ {go_1,go_2, \dots ,go_k} \right\} \ \end{aligned}$$

(ii) According to the tree structure of GO, we can calculate all parental annotation sets of protein *a* under different categories, then add to the original annotation set of GO(*a*), and remove the redundant data. The function annotation of protein *a* obtains as follows.

15 $$\begin{aligned} GO_{sum}(a) = \frac{{GO_{BP_{sum}}(a) + GO_{MF_{sum}}(a) + GO_{CC_{sum}}(a)}}{3}\ \end{aligned}$$

### Cross-validation method

We adopt Cross-Validation to adjust the parameters reasonably under the condition of moderate source datasets and apply them to practical problems. Furthermore, we use the K-fold Cross-Validation method to evaluate the experimental results.

Firstly, we divide the identified host protein complexes into a training set and validation set according to the ratio of 8:2. To know how many validation data are in top *k*, we conduct ten groups of control experiments to verify the results and use the ratio as the final target proteins classification standard. We consider deleting the candidate protein with zero scores in the final ranking process to reduce the data redundancy. Moreover, *k* ranges from 0 to 100 with a step of 10, The Cross-Validation shows that the prediction results can be divided into four categories, as shown in Table 3.

**Table 3:** Classification table of predict results

Type of host protein	Result (whether host protein)	Type of evaluation
Host protein	Positive	TP
Host protein	Negative	FN
Not host protein	Positive	FP
Not host protein	Negative	TN

In this section, the true-positive rate (TPR) and false-positive rate (FPR) values are obtained according to the four results in Table 3, as shown in Eqs. (16) and (17), TPR indicates the prediction coverage of our method.

16 $$\begin{aligned} TPR = \frac{{TP}}{P}\ \end{aligned}$$

17 $$\begin{aligned} FPR = \frac{{FP}}{N}\ \end{aligned}$$

*N* represents candidate proteins that are not in the validation data. *P* represents candidate proteins that are in the validation data. *TP* means the recognized correctly by CCA-SE. *FP* means the recognized quantity wrongly.

Considering the unbalanced data distribution between known and unknown proteins, we use the values of receiver operating characteristic (ROC) and area under the receiver operating characteristic curve (AUC) as evaluation indexes, then plot the ROC curves under different thresholds of TPR and FPR. The abscissa represented the FPR while the ordinate represented the TPR.

## Experiments

### Selection of parameter *k*

**Fig. 3:**
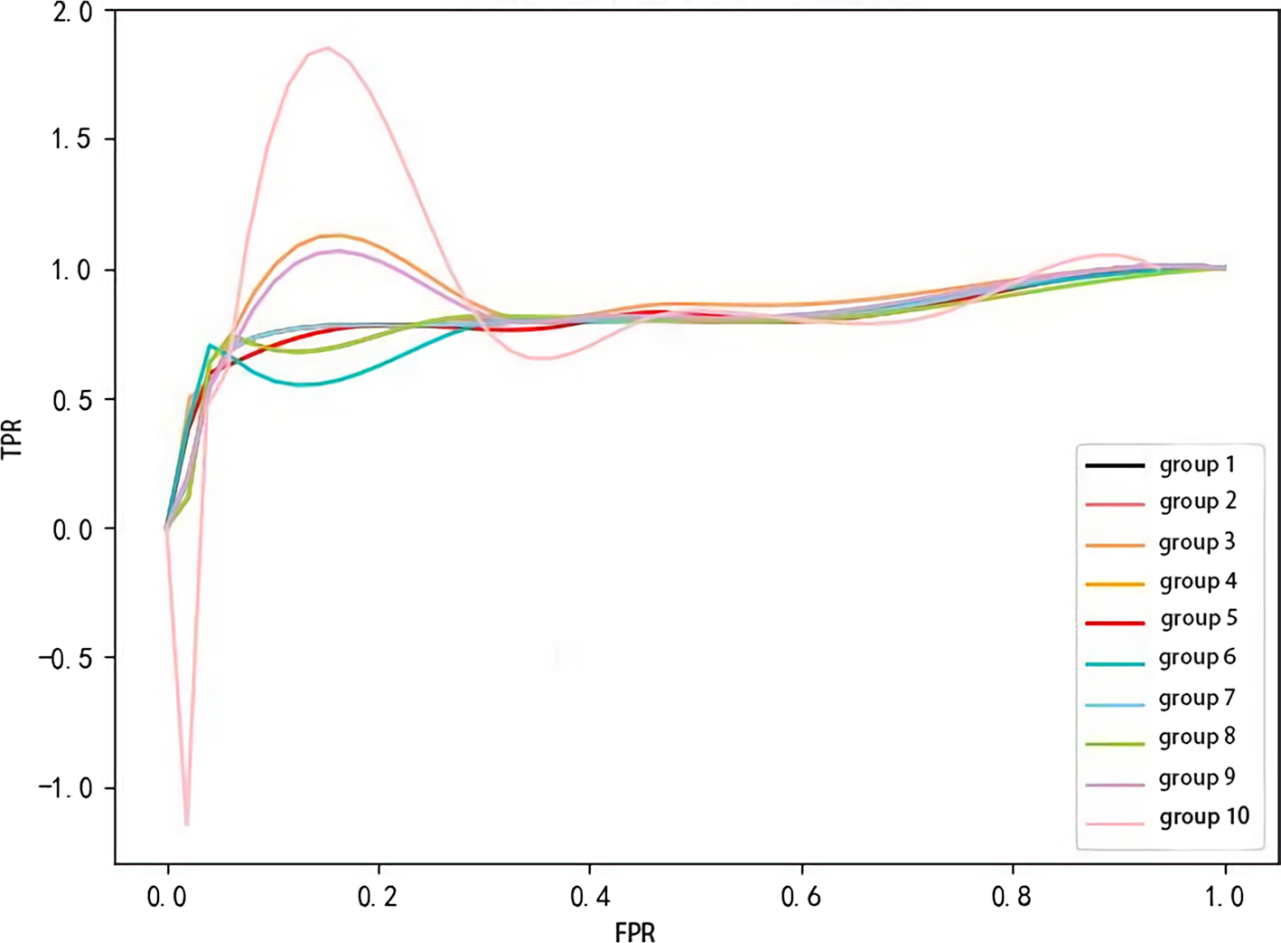
The influence of parameter *k*

**Fig. 4:**
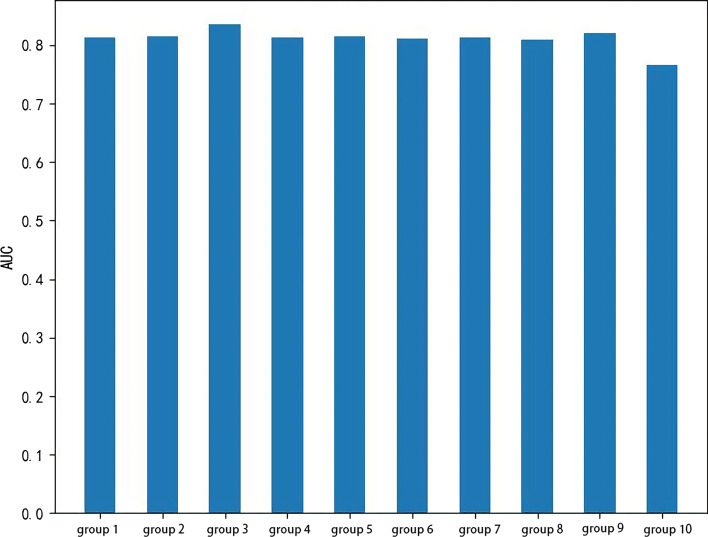
Comparison of AUC values in multiple control experiments

We analyze the influence of parameter *k*, then select the best *k* value. Let the value of *k* vary from 0 to 100. The ROC curve shows in Fig. 3. The prediction results divide into two categories, the first *k*% data considered as the predicted potential target proteins, and the second (100-*k*)% data not considered as the predicted potential target proteins. It can be seen from Fig. 3 that when *k* is 40, CCA-SE successfully predicts 22 known host proteins. According to the above host PPI network, 80% of the proteins are non-structural protein interactions encoded by the 2019-nCov. In addition, this experimental result also shows that the predicted protein scores are high when the value of *k* increases, indicating that the use of Eq. (7) helps to improve the sorting performance of candidate proteins. We plot the AUC curve to show the advantages and disadvantages of each group of algorithms. As shown in Fig. 4, the AUC curve is relatively stable in the ten groups of control experiments, which is basically around 0.81, indicating that the algorithm still shows good performance even when the amount of data is less than the actual biological network data.

In summary, in the subsequent prediction of potential virus–target proteins, we set the value of *k* as 40 to achieve the best experimental results.

### Comparative experiment of data integration

To make a horizontal comparison and evaluate the applicability of the integrated data [32], we compare the results of adding complexes data based on CCA-SE with only containing public human protein complexes data set. We also use TPR and FPR as evaluation indicators. The following table shows that the TPR containing public human protein complexes is only 68.67%, which indicates that the integrated protein complexes data can improve accuracy and make the biological data more comprehensive. It also shows that the complexes recognized by CCA-SE are more biological (Table 4).

**Table 4:** Experimental comparison of whether the complex is integrated or not

Comparision algorithm	Number of recognition	TPR (%)	FPR (%)
Unintegrated data	17	68.67	38.08
Integrated data	22	72	40.31

### Performing GO enrichment analysis on prediction results

After we obtain biological data, to read the genetic information, differential genetic analysis is a necessary experiment between different samples. Therefore we need to annotate these genes because the number of these genes are maybe large and difficult to compare. A common method divides these genes or proteins into several categories, one category is equivalent to a GO term. This process is called enrichment analysis. Commonly used enrichment analysis methods include GO analysis and KEGG analysis.

We integrate known virus–target host proteins with their directly interacted human proteins and apply CCA-SE to the whole network. Our predicted results are listed in “Additional file 1” and named “new targets”. In our results, eight proteins have been proved among the top ten, which are Q13617, Q15370, P62877, Q15369, P09884, Q14181, P35658, and P78406. It reported that 87% of them combined with a virus of non-structural proteins. At the same time, these non-structural proteins are cleaved by 3CLPro, and this protease is one of the organic substances necessary for the reproduction of 2019-nCov. On the other hand, researchers have not recognized similar restriction sites in the human body. It is a medical value that we target 3CLPro as a drug target. Based on the above analysis, CCA-SE can recognize virus–target host proteins. Table 5 is part of the potential target proteins.

**Table 5:** Some of the potential virus–target host proteins

Uniprot ID	Protein name	MF	CC	BP
O15264	Mitogen-activated protein	GO:0005515;	GO:0005829;	GO:0071347;
	Kinase 13 (MAPK13)	GO:0005524;	GO:0005737;	GO:0018105
		GO:0004674;	GO:0005634;	GO:0070301;
		GO:0004707;		GO:0007049;
				GO:0050729;
				GO:1903936;
				GO:0006970;
				GO:0051403;
Q92598	Heat shock protein family H	GO:0005515;	GO:0005654;	GO:1900034;
	(Hsp110) member 1 (HSPH1)	GO:0005524;	GO:0005829;	GO:0051135;
		GO:0000774;	GO:0005737;	GO:0045345;
		GO:0043014;	GO:0005634;	GO:0051085;
			GO:0070062;	GO:0006986;
			GO:0032991;	GO:0050790;
			GO:0071682;	GO:0006898;
			GO:0005874;	
			GO:0005576;	
Q13177	p21 (RAC1) activated kinase	GO:0005515;	GO:0005829;	GO:0071407;
	2(PAK2)	GO:0042802;	GO:0005737;	GO:0050770;
		GO:0019901;	GO:0005634;	GO:0051497;
		GO:0005524;	GO:0098978;	GO:0018105;
		GO:0045296;	GO:0005911;	GO:0031295;
		GO:0004674;	GO:0014069;	GO:0040008;
		GO:0030296;	GO:0048471;	GO:0043066;
		GO:0031267;	GO:0005886;	GO:0006469;
		GO:0004672;		GO:0150105;
				GO:0034333;
				GO:0006468;
				GO:0046777;
				GO:0050852;
				GO:0031098;
Q15311	ralA binding protein	GO:0005515;	GO:0005829;	GO:0043547;
	1(RALBP1)	GO:0042910;	GO:0016020;	GO:0007264;
		GO:0005096;		GO:1990961;
		GO:0042626;		GO:0051056;
		GO:0022857;		GO:0043087;
				GO:0006935;
				GO:0006897;
				GO:0055085;
P78545	E74 like ETS transcription	GO:0005515;	GO:0005654;	GO:0045892;
	Factor 3(ELF3)	GO:0000978;	GO:0005829;	GO:0045944;
		GO:0001228;	GO:0005634;	GO:0006366;
		GO:1990837;	GO:0005794;	GO:0045747;
		GO:0003700;		GO:0006357;
		GO:0000981;		GO:0030855;
				GO:0060056;
				GO:0006954;
				GO:0001824;
				GO:0030198;
				GO:0030154;

To verify the accuracy of the CCA-SE in predicting candidate virus–target proteins, we only conduct gene enrichment analysis for the top 50 potential target proteins. Table 6 lists the enrichment analysis results of proteins that are high scores. These GO terms meet the *p* value of less than 0.05. The analysis results in Table 6 showed that most predicted GO annotations of target proteins are related to biological processes such as protein binding, enzyme binding, transcription factor binding, transcription factor activity, protein kinase binding, apoptosis, and proliferation. For example, GO:0005515, which belongs to MF. Previous studies have found that the spike protein RBD encoded by 2019-nCov contains six amino acids, including L455, F486, Q493, S494, N501, and Y505. Meantime, RBD can integrate with the ACE2 protein of human lung epithelial cells, so we can infer that the host protein corresponding to this functional annotation has a huge correlation with these residues. Another example, GO:0016032, which plays an important role in virus affection, relates to the viral genome replication and the assembly of progeny virus particles. Moreover, Babak Khorsand [18] listed the most central nodes in human interactions of 2019-nCov in his paper, which are Q86VP6, Q92905, Q13573, and P01106. Our results included Q92905, Q13573, and P01106, and we both performed experiments in the same datasets. The prediction of 2019-nCov-target potential host proteins shows a significant enrichment effect. Demonstrating the accuracy of our prediction based on a molecular network.

**Table 6:** Results of GO enrichment analysis

GO ID	GO terminology	*p* value
GO:0016032	Viral process	1.23e−4
GO:0003677	DNA binding	1.40e−4
GO:0004842	Ubiquitin-protein transferase activity	1.32e−4
GO:0005515	Protein binding	1.33e−4
GO:0031625	Ubiquitin protein ligase binding	1.09e−05

### KEGG pathway analysis of prediction results

Kyoto Encyclopedia of Genes and Genomes (KEGG) is a database with functional information about each gene. The core of the KEGG database is KEGG PATHWAY and KEGG ORTHOLOGY. In KEGG PATHWAY, biological metabolic pathways are divided into six categories, namely Cellular Processes, Environmental Information Processing, Genetic Information Processing, Human Diseases, Metabolism, and Organismal Systems. Once we get the differential gene information, in order to learn their functions more clearly, gene enrichment analysis may be used to discover biological pathways that play a key role in biological processes, so that we can better understand the molecular mechanisms of biological processes. KEGG pathway analysis [33] selects pathway databases and human-related pathways to analyze the predicted proteins.

**Fig. 5:**
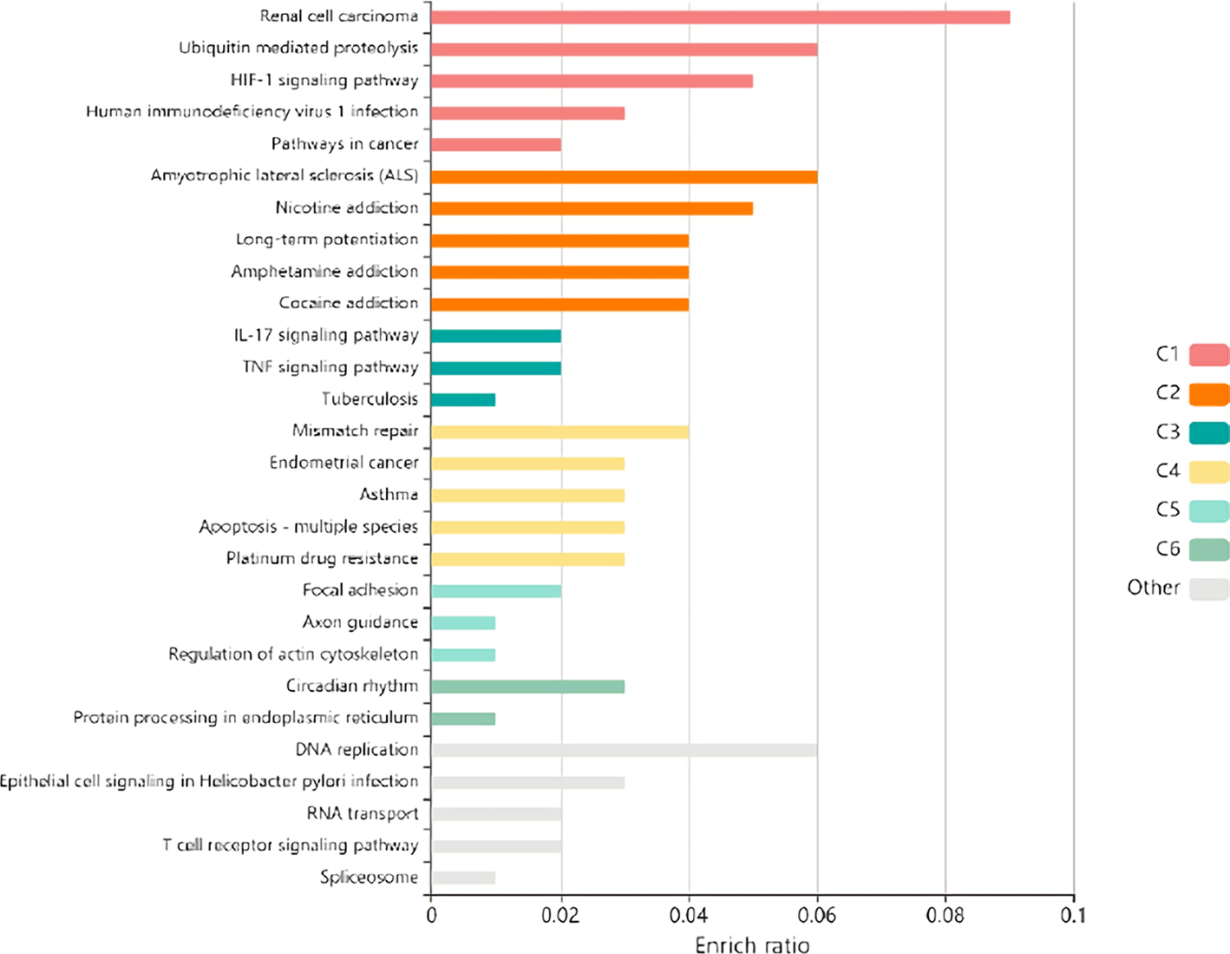
Enrichment function analysis of KEGG signaling pathway

In Fig. 5, C1–C6 represents the result processed by KEGG pathway enrichment analysis. C1 means Annotation Cluster 1, C2 means Annotation Cluster 2, and so on. We sort the results in descending order of Enrichment Score, and “other” includes those genes that do not belong to any of the clusters. These genes have not shown their functional characteristics in our pathway analysis, we consider them less important factors in our potential virus–target experiments. According to Fig. 5, a total of 42 pathways (p-value 0.05) are obtained by screening proteins with high scores, mainly including the TNF signaling pathway, T cell receptor signaling pathway (TCR) pathway, and MAPK pathway related to cell cycle and inflammatory immune regulation, PI3K-Akt signaling pathway and HIF-1 signaling pathway related to pulmonary fibrosis regulation, renal cell carcinoma pathway related to viral diseases and human immunodeficiency virus 1 infection pathway. The above pathways demonstrate that although some predicted host proteins do not directly interact with virus-encoded proteins, they are closely related to the pathogenesis of the virus.

The existing studies have shown that the MAPK pathway is related to cell growth and mutations. TNF is mainly produced by T cells and NK cells, and both are closely related to inflammation. They can release signals to interact with specific receptors on the cell surface, making them conservative, so that MAPK-JNK, 5-lipoxygenase, and other signaling pathways are activated, making cytokines related to inflammation disorders, such as abnormal expression of gp130 and IL-1, and promoting human inflammatory response [3, 4].

**Fig. 6:**
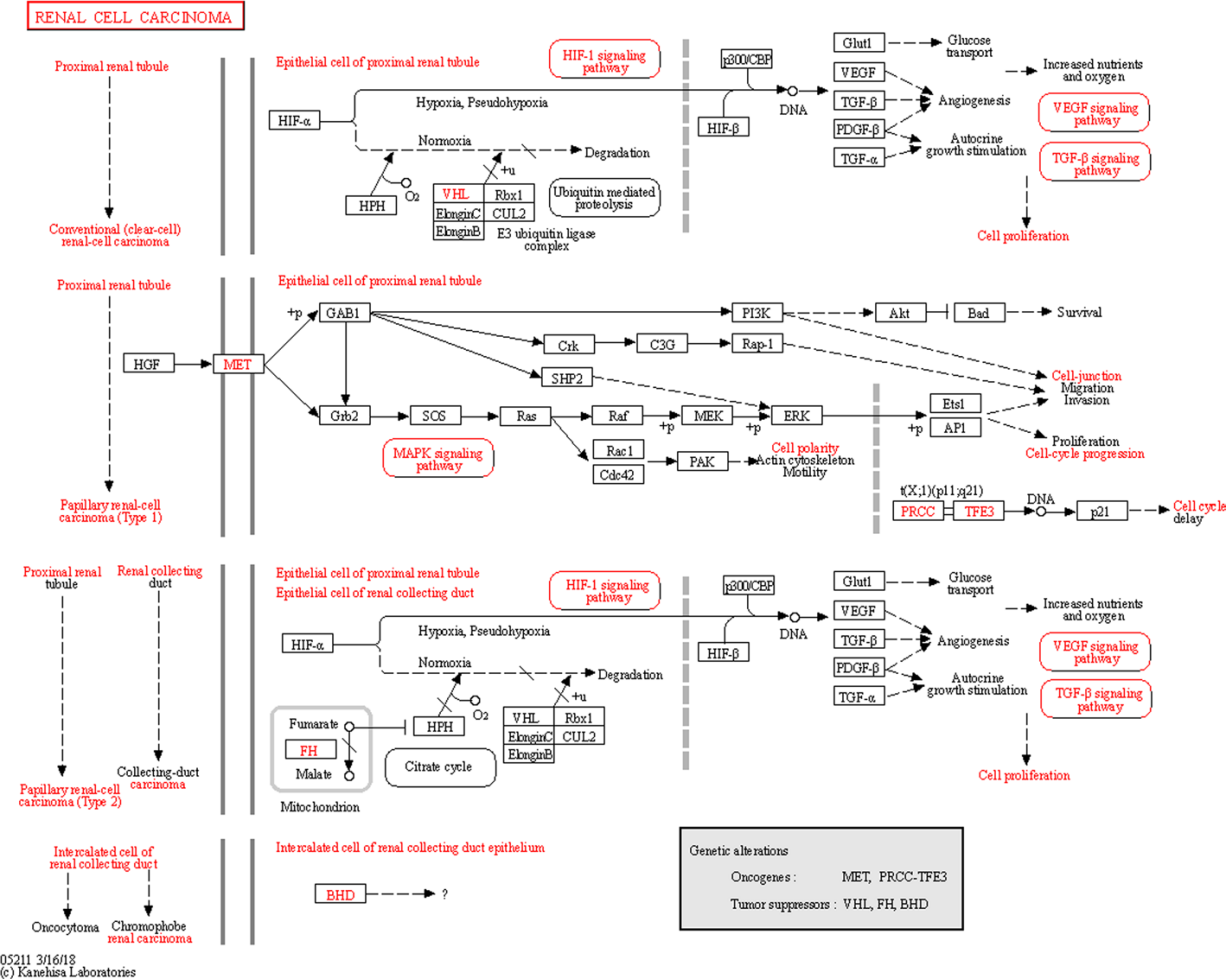
Renal cell carcinoma pathway

The PI3K-Akt signaling pathway is closely related to the renal cell carcinoma pathway, as shown in Fig. 6. Tyrosine kinase receptors can activate phosphatidylinositol 3-kinases (PI3Ks) signaling pathways, which are related to cell proliferation and apoptosis. When PI3Ks is activated, it produces a messenger that binds to the signal protein PDK1 containing the PH domain. Through phosphorylation, the Akt signaling protein is activated to form PI3K-Akt. This protein can also phosphorylate and regulate the downstream factor mammalian target of rapamycin (mTOR) [34], thereby activating the mTORC1 pathway, which participates in the expression of T cytokines in the immune system and promotes the enhancement of the immune system.

## Conclusion

In this paper, we proposed a protein complexes recognition method CCA-SE. The protein complexes obtained by CCA-SE were integrated with the human protein complexes to obtain a more reliable protein complexes dataset, then we defined a score function to get potential target proteins. The scoring function takes into account not only the relationship between the protein complexes and the virus-encoded proteins but also the protein itself to predict the virus–target human proteins. Moreover, we verified the effectiveness of CCA-SE on the biological network under different parameter settings. At the same time, the selected target proteins were imported into the DAVID v6.7 database (https://david.ncifcrf.gov/). We conducted GO function enrichment analysis and KEGG signal pathway enrichment analysis. The analysis explained the correlation between the predicted results obtained by the CCA-SE and the life process of virus infection and replication and proved the accuracy from the biological perspective. The experimental results showed that CCA-SE can effectively recognize human proteins targeted by the 2019-nCov and play a fundamental role in detecting virus drug targets.

## Supplementary Information

**Additional file 1.** The Additional file 1 contains “new targets”. “new targets” lists our predicted results and shows our method of predicting virus–target human proteins does achieve good results.

## Data Availability

The dataset supporting the conclusions of this article is included within additional file. All the experimental code and data can be downloaded from https://github.com/LittleBird120/DiseaseGenePredicition.

## Abbreviations

2019-nCov: The novel coronavirus
PPI: Protein–Protein Interaction
ECC: Edge clustering coefficient
GO: Gene Ontology
CCA: Coverage clustering algorithm
BP: Biological process
MF: Molecular function
CC: Cellular component
TPR: The true positive rate
FPR: The false positive rate
ROC: Receiver Operating Characteristic
AUC: Area under the Receiver Operating Characteristic
KEGG: Kyoto Encyclopedia of Genes and Genomes
TCR: T cell receptor
PI3Ks: Phosphatidylinositol 3-kinases
mTOR: Mammalian target of rapamycin
